# Transcriptional condensates and the nuclear pore complex regulate gene expression and 3D genome architecture in response to stress

**DOI:** 10.1042/BST20253086

**Published:** 2025-10-16

**Authors:** Suman Mohajan, David S. Gross

**Affiliations:** Department of Biochemistry and Molecular Biology, Louisiana State University Health Sciences Center, Shreveport, LA 71130, U.S.A.

**Keywords:** cell nucleus, chromatin, genome integrity, heat shock, nuclear pores, protein misfolding, transcription

## Abstract

Cells respond to thermal, chemical, and oxidative stress by activating an evolutionarily conserved adaptive mechanism known as the heat shock response (HSR) that maintains protein homeostasis and ensures cell survival. Central to the HSR is Heat Shock Factor 1 (HSF1), a highly conserved master transcription factor that up-regulates genes encoding molecular chaperones and other homeostasis factors in response to proteotoxic stress. In both yeast and mammals, the HSR is accompanied by the inducible formation of phase-separated condensates that concentrate components of the transcriptional machinery into discrete intranuclear foci. The assembly of these condensates may be driven by a combination of liquid–liquid phase separation and low-valency Interactions with spatially Clustered Binding Sites (ICBS). In budding yeast, these condensates – which contain HSF1, Mediator, and RNA polymerase II – drive concerted intraand interchromosomal interactions between HSF1 target genes, creating extensive DNA loops between regulatory and transcribed sequences. In this and other ways, yeast *HSR* genes resemble mammalian super-enhancers. Emerging evidence suggests that the nuclear pore complex (NPC) – a macromolecular assembly at the nuclear periphery that regulates protein and RNA transport across the nuclear membrane – serves as a scaffold for the formation of transcriptional condensates and maintains chromatin architecture. In yeast, nuclear basket proteins – which dynamically exchange between the NPC and nucleoplasm – contribute to the heat shock-induced intergenic clustering of HSF1 target loci, whereas essential NPC scaffold-associated proteins do not. Such gene clustering is accompanied by the formation of multiplexed *HSR* mRNAs that could potentially co-ordinate both mRNA export and translation. Here we review evidence that links genome architecture, transcriptional condensates, the NPC, and nuclear basket proteins and discuss potential implications for the treatment of disease.

## Introduction

Cells respond to stress through diverse mechanisms that are critical for maintaining homeostasis and ensuring survival. Under thermal, chemical, or oxidative stress, cells activate the evolutionarily conserved heat shock response (HSR) to maintain protein homeostasis (proteostasis) [1,2]. The longstanding model for HSR activation invokes toxic protein aggregates formed by partially denatured or misfolded proteins due to exposure to stress as the key activators of this response. However, recent findings in budding yeast suggest an alternative model wherein ‘condition-specific adaptive biomolecular condensates,’ composed of orphan ribosomal proteins and stress granule components, act as principal activators of the HSR (reviewed in [3]).

The HSR is characterized by a gene regulatory network that controls the expression of genes encoding molecular chaperones – commonly referred to as heat shock proteins (HSPs) – and other homeostasis factors [1,3,4]. Here, we collectively refer to these genes, whose transcription is under the control of Heat Shock Factor 1 (HSF1), as *HSR* genes. HSF1 is an evolutionarily conserved transcription factor (TF) that inducibly binds to cognate sequence motifs known as heat shock elements (HSEs) located upstream of its target genes [5–7]. Under non-stress conditions, HSF1 is held in an inactive state by the molecular chaperone HSP70 and its co-chaperone HSP40 in an HSF1-HSP70-HSP40 inhibitory complex. Following acute heat shock, HSP70 and HSP40 dissociate, allowing HSF1 to trimerize and bind the HSE motif, thereby activating transcription of *HSR* genes [3,8–10]. Once the cell restores proteostasis, HSP70 re-associates with HSF1 and attenuates the HSR through a negative feedback loop [11].

Recent studies in human, mouse, and yeast have demonstrated that HSF1 forms reversible subnuclear clusters at *HSR* gene loci upon heat shock [12–14]. These HSF1 clusters co-phase separate with the transcriptional machinery – including RNA polymerase II (Pol II) and Mediator – to form transcriptional condensates, here termed ‘HSR condensates.’ These HSR condensates are typically located at active sites of *HSR* gene transcription ([12,13]; reviewed in [3]); although their presence does not always lead to transcriptional activation [15]. These heat shock-inducible HSR condensates drive the dynamic reorganization of the yeast genome [12], a restructuring that leads to *cis-* and *trans*-intergenic interactions between *HSR* genes dispersed across multiple chromosomes [12,15–17]. This clustering of *HSR* genes has been likened to a primordial super-enhancer (SE) as *HSR* gene clusters structurally and functionally resemble mammalian SEs [18].

The function of SEs goes beyond transcriptional induction. Recent studies indicate that SEs acquired during oncogenesis interact with the nuclear pore complex (NPC) to facilitate the rapid export of transcripts, preventing their degradation in the nucleus [19,20]. The NPC is a multiprotein structure located at the nuclear envelope that regulates the trafficking of macromolecules between the nucleus and cytoplasm. Besides their canonical transport functions, the NPC and its component nucleoporins (Nups) also facilitate the assembly of transcriptional complexes and/or transcriptional condensates to enhance gene expression [21–25]. Furthermore, the NPC acts as a scaffold for chromatin architectural proteins and contributes to the spatial organization of chromatin [19,22,26]. In yeast, inducible genes, including a subset of *HSR* genes, relocate to the NPC upon their transcriptional activation [27–30]. This repositioning correlates with the regulation of gene expression, co-transcriptional recruitment of mRNA export factors, and repair of DNA damage [28,29,31–33]. Recent studies in yeast and mammals reveal that heat shock-inducible *HSR* mRNAs cluster together into ribonucleoprotein particles (RNPs) that may promote their preferential export through the NPC [34–36]. The biological significance of HSR condensates, therefore, may extend beyond the transcriptional control of *HSR* genes, as such structures may foster posttranscriptional control or preferential mRNA export or efficient translation. In this review, we explore the structure and function of the NPC, the formation and function of transcriptional condensates, and the interplay between them to gain insight into the molecular mechanism of gene regulation during the HSR and the potential relevance of the NPC and the processes it regulates to human disease.

## Structure and multifaceted role of the NPC

### NPC structure and components

**Figure 1:**
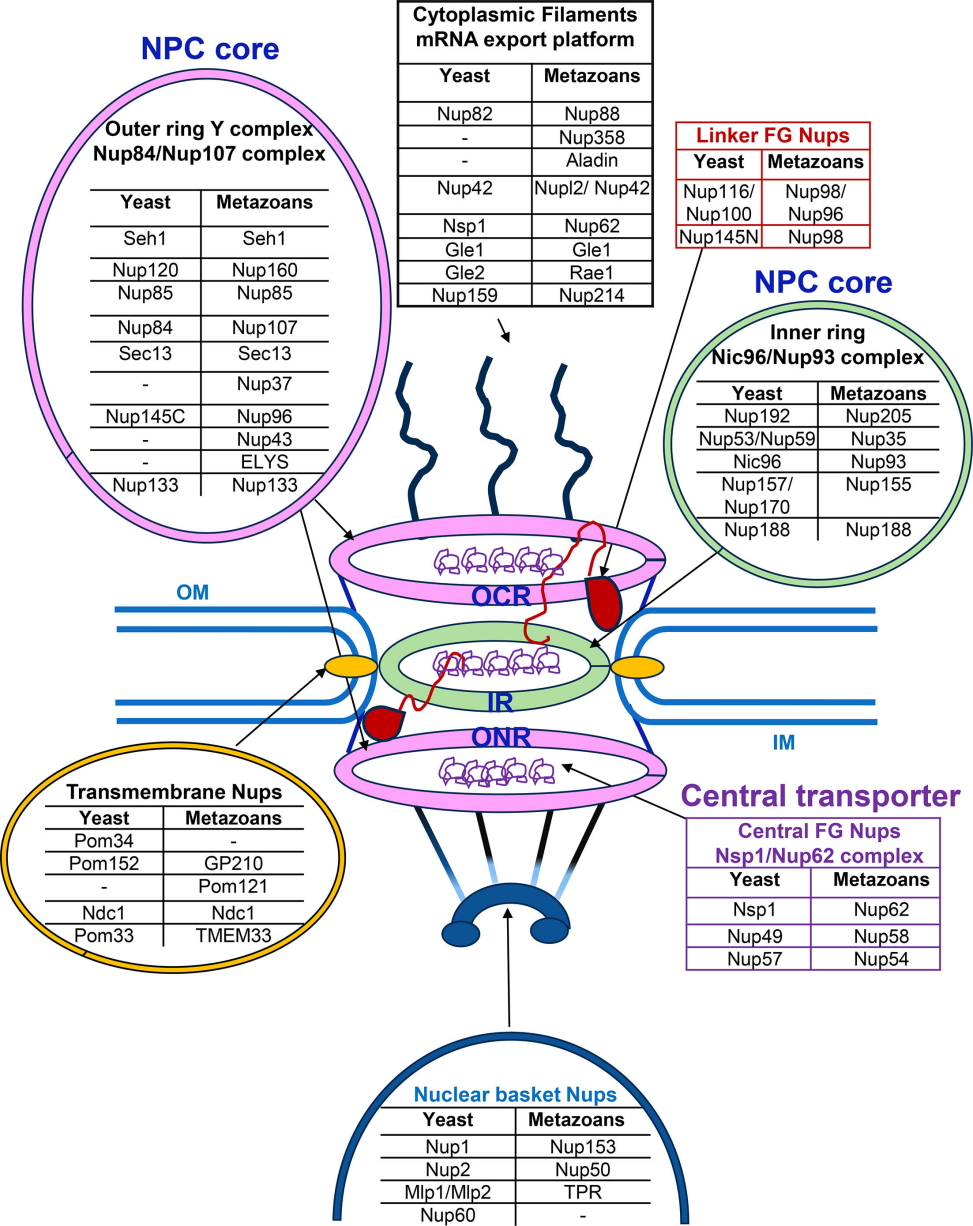
Nuclear pore complex structure. Illustrated are components of the NPC, termed nucleoporins (Nups), conserved from yeast to metazoans [37–39]. Nups are organized into different subcomplexes that form the framework of the NPC. These subcomplexes are color-coded in this figure. The NPC core is composed of two outer rings and one inner ring. The outer ring / Y complex / Nup84/Nup107 complex is depicted in pink; cytoplasmic filaments (mRNA export platforms) are shown in black; Inner ring/Nic96/Nup93 complex is in green; transmembrane Nups are in yellow; Central Transporter is in purple; Nuclear Basket is in blue; and linker Nups are in red. ELYS, embryonic large molecule derived from yolk sac; FG, Phe-Gly; Gle, GLFG (Gly-Leu-Phe-Gly) lethal; GP210, glycoprotein 210; IM, inner membrane; IR, inner ring; Mlp1/Mlp2, myosin-like protein 1/2; Ndc1, nuclear division cycle protein 1; Nic96, Nup-interacting component of 96 kDa; Nsp1, nucleoskeletal-like protein 1; Nupl2, nucleoporin-like 2 protein; OCR, outer cytoplasmic ring; OM, outer membrane; ONR, outer nuclear ring; Pom, pore membrane protein; Rae1, ribonucleic acid export 1; Seh1, SEC13 homologue 1; TMEM33: transmembrane protein 33; TPR, translocated promoter region.

Each NPC consists of multiple copies (8–64) of around 30 different types of proteins known as nucleoporins (Nups), ~500–1000 in total, which serve as the fundamental building blocks of the NPC. Most are conserved across eukaryotes, as is the overall structure. The NPC consists of two distinct substructures: the central core structure, which is embedded between the inner and outer nuclear membranes, and peripheral structures, which extend from the central core toward the cytoplasmic and nucleoplasmic sides (Figure 1; central core labeled outer cytoplasmic ring [OCR], inner ring [IR], and outer nuclear ring [ONR]). The central core structure features an eightfold rotational symmetry that surrounds a central tube (the latter referred to as the ‘Central Transporter’). The core structure includes outer rings on both the cytoplasmic side (OCR) and the nucleoplasmic side (ONR) of the NPC and an IR sandwiched between the two outer rings. The outer rings are anchored to the core by the IR (reviewed in [37,38]). The human NPC has double nuclear rings, while yeast NPCs exhibit a mix of single and double nuclear rings [40]. In actively growing yeast cells, ~70% of NPCs possess a single nuclear ring, while the remainder have double nuclear rings [40]. The peripheral structure includes cytoplasmic filaments and a nuclear basket that emanate from cytoplasmic and nuclear rings, respectively. Cytoplasmic filaments contain intrinsically disordered regions (IDRs) and are highly flexible, allowing them to interact with cargo. The nuclear basket functions as a docking site for mRNP granules because of its flexibility; it also plays a role in mRNA metabolism. The NPC core structure is anchored to the nuclear membrane via transmembrane Nups. Finally, the NPC central transporter, lined with highly disordered Phe-Gly repeat-containing Nups, serves as a route for bidirectional nucleocytoplasmic transport (reviewed in [37–39]).

### Nucleoporins: beyond nucleocytoplasmic transport

#### Chromatin organization

The canonical function of the NPC is to mediate selective nucleocytoplasmic transport. Beyond this transport capability, the NPC also plays a crucial role in maintaining chromatin organization. Chromatin that is in close proximity to the NPC is observed to be less condensed compared with other locations along the nuclear periphery [41]. The exclusion of condensed heterochromatin from the nuclear pore is facilitated by the basket Nup known as TPR in human, Megator in Drosophila, and myosin-like protein 1/2 (Mlp1/2) in yeast [42]. Yeast Mlp1/Mlp2 assemble into coiled-coil dimers that comprise key components of the NPC basket (see Figure 1); these proteins also extend horizontally to link adjoining NPCs. They help keep the area beneath the NPC central tube free from dense, repressive chromatin [43].

The basket nucleoporin Nup2 similarly acts as a boundary element or insulator that prevents the spread of heterochromatin towards more active loci by directly or indirectly tethering to chromatin [44,45]. In addition to these boundary roles, yeast Nups also mediate the spatial repositioning of transcriptionally induced genes from the nuclear interior to the nuclear pore, and genes with similar upstream regulatory sequences termed ‘zip codes’ undergo interallelic clustering at the NPC [46,47]. Likewise, several studies in fly and mammalian cells revealed that Nups mediate long-range chromatin interactions by directly associating with enhancers, promoters, and architectural proteins [26,48]. These interactions predominantly occur at the NPC, suggesting that the NPC serves as a scaffold for the spatial organization of chromatin [19,22,26]. The NPC in budding yeast has also been implicated in the global reorganization of chromatin in response to various physiological stimuli [49]. We discuss further the roles of the NPC in mediating long-range chromatin structure and transcriptional regulation below.

#### Transcription regulation

Research across various model systems has shown that Nups physically interact with many chromosomal loci, regulating their spatial organization and overall gene expression [27,50,51]. For example, in budding yeast, Nups comprising the inner and outer rings of the NPC (i.e. Nup170 and Nup145), along with nuclear basket (Mlp1 and Mlp2) and nuclear envelope proteins, tether telomeres and adjacent sequences to the periphery. This anchoring promotes recruitment and stabilization of the Rap1/Sir2/Sir3/Sir4 silencing complex at the chromatin, thereby silencing the expression of genes tethered to the nuclear periphery ([52,53]; reviewed in [54]). Similarly, in Drosophila, interaction between the NPC core nucleoporin Nup93 and the Polycomb domains contributes to the repression of Polycomb target genes [55].

 Nups and chromatin interactions are also implicated in the transcriptional activation of genes through both on-pore and off-pore mechanisms. For instance, in flies, mammals, and yeast, interaction of genes with the NPC and its constituent Nups enhances transcriptional activity and increases gene expression rates [29,56–58]. NPC-gene interactions also contribute to the development of transcriptional or epigenetic memory that promotes faster and more robust reactivation of the genes following reinduction [26,59,60]. Docking of genes at the pore facilitates the recruitment of splicing machinery for efficient mRNA processing [61], suggesting that the NPC co-ordinates transcriptional regulation and mRNA metabolism.

#### DNA damage response (DDR)

NPCs play a crucial role in the DNA damage response (DDR) by acting as hubs that co-ordinate DNA repair activities. It is notable that certain DNA lesions, such as R loops, persistent double-strand breaks (DSBs), collapsed replication forks, and eroded telomeres, relocate to NPCs [33,62,63]. Such relocation facilitates recruitment of DNA repair factors to damaged DNA sites, promoting efficient repair processes such as homologous recombination and non-homologous end joining that ensure the integrity of the genome [64–66]. While involvement of the NPC in the DDR was first observed in yeast, these functions are also conserved in mammals and flies. For instance, in mammalian cells, nuclear F-actin mediates the relocalization of heterochromatic DSBs or telomeres upon replication stress to the nuclear pore and/or periphery [67,68]. In interesting contrast, neither monomeric nor filamentous actin contributes to the 3D spatial relocalization of thermal stress-activated *HSR* genes that culminates in their physical clustering in budding yeast [69]. Human Nup153 (ortholog of yeast Nup1) is required for DNA DSB repair and DNA damage checkpoint activation [70]. Mutations in Nups result in the accumulation of damaged DNA [31,71]. Accumulation of damaged DNA has been implicated in aging and various diseases such as neurodegeneration and cancer [72–74]. These observations highlight the importance of NPCs in maintaining genome integrity and in contributing to genomic transactions beyond transcription.

## Transcriptional condensates: formation and function

Transcriptional condensates are dynamic, membrane-less nuclear compartments that concentrate transcription-related molecules such as transcription factors (TFs), RNA Pol II, and co-activators at specific genomic loci. This compartmentalization of transcription machinery contributes to the robust expression of genes associated with cell identity, proliferation, development, and stress responses [12,13,75–80]. The mechanisms underlying the formation of transcriptional condensates remain enigmatic, although it has been proposed that the phenomenon often involves liquid–liquid phase separation (LLPS).

### Phase separation and formation of transcriptional condensates

Phase separation is a phenomenon driven by weak multivalent interactions between IDR-containing constituent proteins. Many proteins involved in transcription, including TFs, co-activators, and Pol II, contain IDRs. These regions lack a defined three-dimensional structure and are enriched in specific amino acids, facilitating weak, multivalent interactions leading to a network-like structure that promotes condensation or clusters [78,81]. It is often unclear whether these clusters – visualized by either live or fixed cell microscopy – represent true phase-separated entities. Labeling endogenous proteins followed by super-resolution microscopy revealed that TFs, cofactors such as BRD4, Mediator subunits, and Pol II, along with chromatin, can form discrete droplets at a physiological concentration in living cells. These droplets show the ability to rapidly recover their fluorescence after photobleaching and can fuse into large droplets when they touch each other in living cells [77,78]. This behavior is consistent with those structures being biomolecular condensates – self-organized, membrane-free compartments enriched in specific macromolecules that may or may not be phase-separated [82].

Increasing evidence suggests that condensates can form through mechanisms other than LLPS. For example, condensates can arise from macromolecules that engage in strong, low-valency interactions with spatially clustered binding sites (ICBS) on a chromosome segment that act as scaffolds [83–86]. In LLPS, molecules engage in numerous weak multivalent interactions to create a percolated, web-like network that separates into condensed and dilute phases [87,88]. Unlike LLPS, ICBS relies on strong monovalent interactions with binding sites on long-chain polymers and alternations between bound and free states. The bound molecules promote additional molecules to accumulate near the long-chain polymers, leading to condensate formation [85]. Condensates that require DNA or RNA as a scaffold are more likely to form via the ICBS mechanism [86]. Notably, condensate formation does not always occur through a single mechanism, as both LLPS and ICBS mechanisms can coexist or dominate at different stages of condensate formation. For example, in LLPS, protein concentration must exceed a threshold to trigger condensate formation. At lower protein concentrations, DNA scaffold-based ICBS may nucleate condensates, while increasing concentrations can drive a transition from ICBS to LLPS. HSF1-containing nuclear stress bodies (nSBs) in human cells are a relevant example in this case. At low concentration, HSF1 binds to the satellite DNA repeats and initiates the condensation via ICBS. As HSF1 levels rise, condensation accelerates through LLPS [13,86].

It remains unclear whether SE or enhancer hubs drive the formation of transcriptional condensates or transcriptional condensates by themselves drive the formation of enhancer/SE hubs. Recent studies support a model whereby TFs bind to specific genomic loci, such as distal enhancers or promoter-proximal enhancers, and cluster through IDR–IDR interactions (potentially via the ICBS mechanism). Once TFs bind and cluster, they recruit co-activators (Mediator, chromatin-remodeling complexes, etc.), Pol II, and other components of the transcriptional machinery. When the protein concentration exceeds a threshold, large condensates form (potentially via the LLPS mechanism). Condensates that form at a specific enhancer can influence chromatin organization by connecting distant enhancers. This process relies on the coalescence of condensates driven by surface tension, ultimately connecting the constituent enhancers that comprise a SE [89–92].

### Transcriptional condensates and gene activation

Although transcriptional condensates are recognized as key regulators of transcription [77,78,93], the precise functions and mechanisms underlying their action remain unclear. The dynamic kissing model proposes that transcriptional condensates appear at the enhancer and intermittently interact with the promoter of actively transcribed genes, thereby inducing a transcription burst [77,94]. The transcriptional burst size and magnitude depend on the condensates’ proximity and positional dynamics relative to the promoters of active genes [94]. However, transcriptional output also affects the formation and dissolution of condensates. For example, a low level of transcribed RNAs at regulatory elements initially promotes condensate formation. Conversely, a high level of RNA produced during transcription leads to its dissolution through a feedback mechanism [95].

It has been proposed that during transcription, different types of condensates, such as those associated with initiation, elongation, splicing, and termination, regulate distinct phases of transcription. The specific partitioning of biomolecules from one condensate to another depends on the biochemical reactions occurring during transcription. For instance, Pol II carboxy terminal domain (CTD) phosphorylation regulates the partitioning of Pol II between initiation and elongation condensates and between elongation and splicing condensates. Mature RNA is released from Pol II within the termination condensates [96,97]. These features suggest spatial-temporal co-ordination between transcriptional condensates and transcription.

### Transcriptional condensates and gene repression

In addition to serving as activators of transcription, transcriptional condensates may be neutral or inhibitory to transcription [98–100]. A recent study indicates that transcriptional condensates that form at an estrogen receptor target locus prevent the transcription of non-target genes within the same topologically associated domain (TAD) by sequestering transcriptional machinery away from those non-target loci [101]. Similarly, HSF1-containing condensates in nSBs induce transcription of satellite III long non-coding RNA that sequesters critical transcriptional machinery and thereby represses global gene expression [102]. Interestingly, rodents lack this satellite DNA sequence in their genomes and therefore do not form heat shock-induced nSB condensates [103]. This divergence may have provided humans with an additional advantage by enabling a global shutdown of gene expression during stress while preserving transcription of *HSR* genes. Transcriptional condensates may possess an optimal compositional range for achieving maximal activity; however, this optimal range may vary depending on the specific system or conditions. Any surplus or deficiency could inhibit transcription activity [98,104–106].

## Transcriptional condensates that activate the cellular stress response

Transcriptional condensates play a crucial role in cellular stress responses, serving as dynamic hubs for gene regulation and facilitating adaptation to challenging environments. During exposure to thermal or chemical stress, HSF1 orchestrates a rapid and robust cellular response through the formation of HSR condensates [12,13,15]. These intranuclear bodies, which are typically ~300–480 nm in diameter, are to be contrasted with HSF1-containing nSB condensates (≤3 µm) or persistent gel-like HSF1 condensates observed in human cancer [13,102,107]. nSB condensates don’t regulate *HSR* genes but rather induce transcription of long non-coding satellite RNAs that globally suppress transcription [102,108,109] as discussed above, and gel-like HSF1 condensates are an early indicator of apoptosis [107].

### HSR transcriptional condensates and transcription of *HSR* genes

The role of HSR condensates in the transcriptional regulation of *HSR* genes is under active investigation. In budding yeast exposed to acute heat shock, the kinetics of HSR condensate formation and dissolution parallel the transient activation of *HSR* genes [12,15]. Both rapidly increase in cells, peak within minutes, and generally dissipate within one hour [12,15,17]. In contrast, in yeast exposed to 8.5% ethanol, HSR condensates peak very early (within 2.5 min) yet are sustained for hours. In response to this stress, *HSR* transcriptional activation is temporally uncoupled from HSR condensate formation, as it is not detected at most genes until 10–20 min and typically peaks at >1 h [15]. Furthermore, an HSF1 separation-of-function mutant (N-terminal IDR truncation) can activate WT levels of *HSR* gene transcription yet is defective in promoting the formation of HSR condensates [12]. Therefore, in yeast, formation of HSR condensates appears to be neither necessary nor sufficient to drive the transcription of *HSR* genes. By contrast, in human cells, heat shock-induced formation of HSF1-containing condensates drives the robust transcription of *HSR* genes [13].

### HSR transcriptional condensates and the restructuring of 3D genome topology

A novel role attributed to HSR condensates is that they induce dynamic 3D restructuring of the yeast genome in response to proteotoxic stress [12]. This restructuring principally involves members of the HSF1 regulon [16,17]. These condensates drive *HSR* genes – dispersed across multiple chromosomes – into coalesced chromatin clusters. The formation of HSR condensates and 3D spatial repositioning of *HSR* genes exhibit a different temporal relationship in cells subjected to thermal versus chemical stress. In thermally stressed cells, both events occur concurrently; however, in cells exposed to ethanol stress, *HSR* gene clustering is slightly delayed relative to the formation of HSR condensates. Moreover, like HSR condensate formation discussed above, repositioning of *HSR* genes precedes the transcriptional activation of *HSR* genes upon ethanol stress [15]. Collectively, these observations point to different mechanisms by which HSF1-containing condensates orchestrate genome restructuring and gene transcription depending on the nature of the proteotoxic signal.

## Nucleoporins and transcriptional condensates: an integrated model of the heat shock response

### Nucleocytoplasmic transport and heat stress

Heat shock, like other cellular stresses, induces the nucleocytoplasmic redistribution of functional proteins and disrupts conventional transport pathways [110]. For example, heat shock triggers the accumulation and retention of importins in the nucleus, thereby preventing their recycling [111,112]. Conversely, heat shock activates the Hikeshi-mediated nuclear import of the molecular chaperone HSP70 from the cytoplasm [112,113]. Furthermore, heat shock causes activation of the Slt2/Mpk1 MAP kinase pathway and blocks bulk mRNA export while facilitating preferential export of *HSR* mRNA [114–116]. For example, in *Saccharomyces cerevisiae* during acute heat shock, Mlp1 (ortholog of human TPR) dissociates from the nuclear basket and forms intranuclear foci that sequester mRNA export factors such as Nab2 and Yra1 [115,117,118]. Heat shock-induced phosphorylation of Nab2 by Slt2/Mpk1 occurs coincidently with the colocalization of Nab2 with Mlp1 foci. However, it is not clear whether the phosphorylation promotes the formation of Nab2 foci. The sequestration of these export factors results in the retention of bulk poly(A) mRNA within the nucleus, while stress-responsive mRNAs are preferentially exported in a Mex67-dependent manner [115–117]. Similarly, in fission yeast, acute heat shock causes reversible aggregation of nuclear and nucleolar proteins, including NPC proteins, into nucleolar rings (NuRs) that sequester essential factors needed for mRNA metabolism and NPC function. When cells return to their optimal growth temperatures, Hsp70 and the disaggregase Hsp104 dissolve the NuRs, redistributing their sequestered components to their respective functional environments to resume cell growth [119]. Therefore, all these processes indicate multistep regulatory strategies that cells use to survive during acute heat stress.

### Models

#### NPC serves as a scaffold for condensate formation

Recent evidence from studies spanning budding yeast to metazoans supports a model in which the NPC acts as a scaffold for the formation of transcriptional condensates [25,26,120,121]. The NPC and its constituent Nups have been shown to associate with SEs, orchestrating gene regulation through co-ordination with chromatin-modifying enzymes, TFs, and other regulatory proteins [21–25]. Notably, Nups contain IDRs capable of multivalent interactions with IDR-containing components of the transcription machinery, including Med1, Pol II, BRD4, and p300 in mammalian cells [25]. These interactions support NPCs as hubs for assembling macromolecular complexes essential for cell-type-specific transcriptional regulation [25].

The role of Nups as scaffolds for transcriptional condensates is further illustrated by the human Nup98-HOXA9 fusion chimeric TF. The IDRs of NUP98 (comprise tandemly dispersed FG repeats) drive the formation and composition of liquid–liquid phase-separated chromatin-bound onco-condensates [122,123]. These chromatin-bound multicomponent onco-condensates drive the formation of transcriptional hubs or SE-like binding patterns that contribute to aberrant transcription [122,123]. This conceptual framework highlights the dual role of the NPC in nucleocytoplasmic transport and transcriptional regulation, emphasizing its critical function in organizing transcriptional condensates to enable precise control of gene expression.

### Nuclear basket proteins as dynamic participants in genome restructuring

Numerous studies have underscored the critical role of the NPC and its constituent Nups in shaping the 3D organization of the genome. This spatial organization is essential for regulating gene expression linked to developmental processes, cell type differentiation, and disease progression [23,48,122,124]. In budding yeast, NPCs are thought to act as platforms for the repositioning and clustering of inducible genes in response to cellular stress, facilitating co-ordinated transcriptional regulation [46,47,125].

*HSR* genes – such as *HSP104, SSA2,* and *SSA4* – reposition to the nuclear pore in response to acute heat shock through a mechanism dependent on the nuclear basket proteins Nup2 and Mlp1 [28,30]. Ironically, heat shock-induced coalescence of *HSR* genes such as *HSP12, HSP82, HSP104*, *SSA2*, *SSA4,* and *UBI4* primarily occurs within the nucleoplasm, where both Nup2 and Mlp1 – mobile Nups that can bind to chromatin – dynamically contribute to 3D genome restructuring [118].

**Figure 2:**
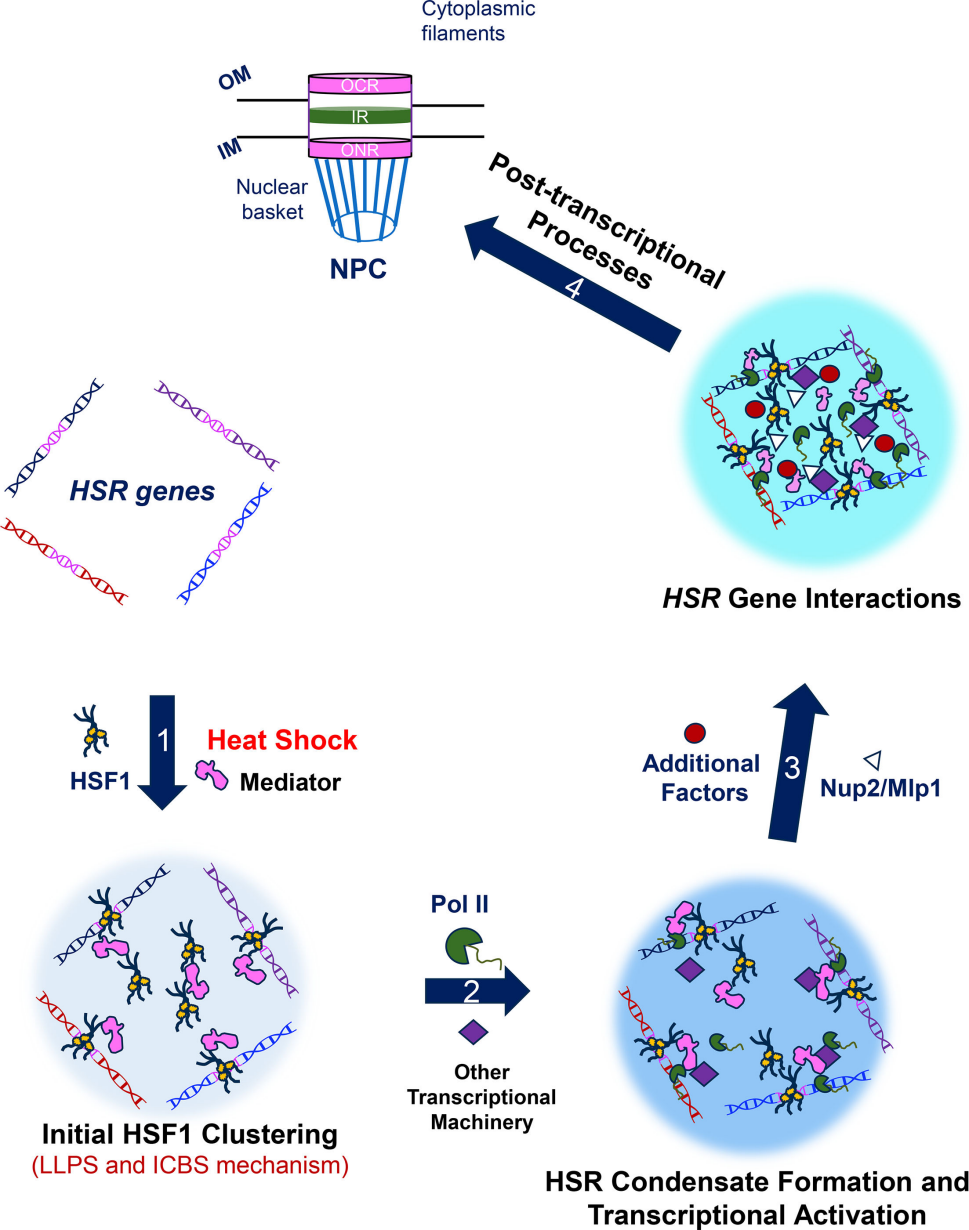
Nuclear basket proteins contribute to the 3D restructuring of *HSR* genes following the formation of HSR condensates. Depicted is a model of events occurring in the nucleus of a budding yeast cell exposed to acute thermal stress. Heat shock induces the activation and binding of HSF1 to HSR elements located upstream of *HSR* genes (HSF1 clustering). Recruitment of HSF1 and Mediator may occur as a concerted event (G. Male, personal communication). Some clusters may exist as clouds over the *HSR* genes [15,100]. Formation of these clusters is likely driven by both LLPS and ICBS mechanisms [12,86]. HSF1 then recruits components of the transcriptional machinery, leading to the formation of HSR condensates [12,15,126], again by both LLPS and ICBS mechanisms. These HSR condensates serve as the precursors for initiating transcription and the 3D reorganization of *HSR* genes. Roughly concurrent with the formation of HSR condensates and activation of *HSR* genes, nuclear basket proteins and other regulatory factors are recruited to these genes. These factors then drive the physical clustering of *HSR* genes – a phenomenon that is predominantly detected within the nucleoplasm [118]. Subsequently, the *HSR* chromatin clusters reposition to the NPC for post-transcriptional events such as mRNA multiplexing [34–36]. Such repositioning and multiplexing may enhance the processing, export, and/or preferential translation of *HSR* mRNAs [116,127,128]. Therefore, perturbation of *HSR* gene coalescence may result in the nuclear accumulation of *HSR* mRNAs.

These nuclear basket proteins show heat shock-dependent relocalization from the nuclear periphery to the interior. In the nucleoplasm, Nup2 and Mlp1 exhibit a diffuse distribution pattern, with a subset of Mlp1 molecules forming distinct foci. The diffuse distribution of these proteins may facilitate *HSR* gene coalescence directly as a consequence of their association with *HSR* loci [118]. The focal pattern of Mlp1 could contribute indirectly by sequestering inhibitors of *HSR* gene coalescence. Notably, Nup2 and Mlp1 are dispensable for the formation of HSR condensates as well as the recruitment of HSF1 and RNA Pol II at the *HSR* genes. Moreover, neither protein shows colocalization with HSR condensates [118]. Nonetheless, these two nuclear basket proteins promote *HSR* gene coalescence. This supports a model in which HSR condensate formation occurs before 3D reorganization of the yeast genome, and nuclear basket proteins act downstream of HSR condensate formation (Figure 2).

## Implications for disease and therapeutic potential

### Nucleoporins and disease

As discussed above, Nups play a crucial role in maintaining chromatin architecture and regulating transcription. Gene fusions involving Nups, such as the chromosomal translocation of NUP98, have been identified in at least 24 different partner genes linked to hematopoietic malignancy [129]. How these fusions drive malignant transformation has been intensively investigated. The NUP98-HOXA9 fusion protein drives leukemia transformation by inducing aberrant chromatin looping between SE-like binding sites and proto-oncogenes. Genome restructuring may arise from *de novo* formation of condensates driven by FG-repeat-containing IDRs of NUP98 [122,123]. The resultant altered 3D chromatin architecture drives excessive oncogene transcription, ultimately leading to cancer [122]. Likewise, the fusion of NUP98 with PHD-finger proteins has been shown to induce leukemic transformation by locking target genes into an aberrantly active state [130]. Additional mechanisms through which Nups may drive cancer progression include altering nucleoplasmic trafficking, modulating signaling pathways, disrupting cell cycle regulation, reshaping the tumor microenvironment, affecting tumor suppressor functions, and regulating translation [131]. Beyond cancer, Nup dysfunction has been linked to numerous cellular and developmental defects, as well as a range of human diseases such as neurological disorders, autoimmune dysfunction, and cardiovascular diseases [132–134].

### Therapeutic targeting of condensate-triggering nucleoporins

Emerging evidence suggests that targeting core components of the condensates or their IDRs to disrupt the molecular forces regulating the formation of transcriptional condensates holds significant promise as a therapeutic strategy for diseases such as cancer. For instance, targeting NUP98 fusion proteins and their associated proteins with inhibitors that prevent chromatin binding, condensate formation, and oncogene expression represents a potential therapeutic approach for patients with leukemias [135–137]. Similarly, the inhibition of NUP153 chromatin binding using epigenetic inhibitors could provide a promising targeted therapy for patients with Duchenne muscular dystrophy [138].

## Current limitations: gaps in knowledge and technical challenges

Research on Nups and transcriptional condensates in the context of the cellular stress response encounters several significant challenges. One major challenge is the complexity of cellular stress responses, which involve multiple signaling pathways and mechanisms. Additionally, the molecular mechanisms that regulate the formation of transcriptional condensates and the reorganization of genome architecture in response to cellular stress are still not well understood.

Many Nups have overlapping roles, and their interactions with various cellular components can create a convoluted picture of their contributions to stress responses. The identification of specific Nups involved in these processes and their precise functions presents another challenge. Furthermore, transcriptional condensates are not static; they constantly undergo formation and dissolution in response to cellular conditions. They exhibit great diversity in biochemical composition and physical properties. Capturing their transient behavior, along with accurately categorizing and functionally analyzing their diversity through currently available experimental approaches, can be challenging and hinder understanding of the underlying molecular mechanisms driving the aforementioned processes in the stress response.

**Perspectives**

- The heat shock transcriptional response (HSR) is a powerful model system to understand the mechanisms underlying gene regulation and genome organization. Elucidating the complex interplay between the nuclear pore complex (NPC) and transcriptional condensates will provide new insights into fundamental cellular mechanisms underlying diseases such as cancer and neurodegeneration.
- Condensation of gene-specific transcription factors and their co-regulators is thought to underlie the activation of many, if not most, protein-encoding genes. The NPC and its constituents act as scaffolds that organize 3D genome structure and establish transcriptional hubs essential for cell-type-specific and oncogene-linked gene regulation. NPCs act as platforms for the repositioning and clustering of inducible genes in some organisms, and in those organisms, Nups comprising the nuclear basket contribute to transcriptional memory.
- Investigating the spatiotemporal interplay between NPCs, transcriptional condensates, and the restructured 3D genome using advanced molecular and imaging approaches could significantly enhance our understanding of the composition, properties, and functions of stress-inducible transcriptional condensates and Nups. Moreover, exploring the effects of specific genetic modifications or drug treatments on Nups and condensate dynamics could lead to the identification of new therapeutic targets.

## Open Access

This article has been published open access under our Subscribe to Open programme, made possible through the support of our subscribing institutions, learn more here: https://portlandpress.com/pages/open_access_options_and_prices#conditional

## Abbreviations

DDR: DNA damage response
DSB: double-strand break
HSE: heat shock element
HSF1: heat shock factor 1
HSP: heat shock protein
HSR: heat shock response
IDR: intrinsically disordered region
IR: inner ring
LLPS: liquid–liquid phase separation
Mlp: myosin-like protein
NPC: nuclear pore complex
NuRs: nucleolar rings
Nups: nucleoporins
OCR: outer cytoplasmic ring
ONR: outer nuclear ring
Pol II: polymerase II
RNPs: ribonucleoprotein particles
SE: super-enhancer
TF: transcription factor
nSB: nuclear stress bodies
